# Obesity Impairs Lymphatic Fluid Transport and Dendritic Cell Migration to Lymph Nodes

**DOI:** 10.1371/journal.pone.0070703

**Published:** 2013-08-12

**Authors:** Evan S. Weitman, Seth Z. Aschen, Gina Farias-Eisner, Nicholas Albano, Daniel A. Cuzzone, Swapna Ghanta, Jamie C. Zampell, Daniel Thorek, Babak J. Mehrara

**Affiliations:** The Division of Plastic and Reconstructive Surgery, Department of Surgery, Memorial Sloan-Kettering Cancer Center, New York, New York, United States of America; Virginia Tech University, United States of America

## Abstract

**Introduction:**

Obesity is a major cause of morbidity and mortality resulting in pathologic changes in virtually every organ system. Although the cardiovascular system has been a focus of intense study, the effects of obesity on the lymphatic system remain essentially unknown. The purpose of this study was to identify the pathologic consequences of diet induced obesity (DIO) on the lymphatic system.

**Methods:**

Adult male wild-type or RAG C57B6-6J mice were fed a high fat (60%) or normal chow diet for 8–10 weeks followed by analysis of lymphatic transport capacity. In addition, we assessed migration of dendritic cells (DCs) to local lymph nodes, lymph node architecture, and lymph node cellular make up.

**Results:**

High fat diet resulted in obesity in both wild-type and RAG mice and significantly impaired lymphatic fluid transport and lymph node uptake; interestingly, obese wild-type but not obese RAG mice had significantly impaired migration of DCs to the peripheral lymph nodes. Obesity also resulted in significant changes in the macro and microscopic anatomy of lymph nodes as reflected by a marked decrease in size of inguinal lymph nodes (3.4-fold), decreased number of lymph node lymphatics (1.6-fold), loss of follicular pattern of B cells, and dysregulation of CCL21 expression gradients. Finally, obesity resulted in a significant decrease in the number of lymph node T cells and increased number of B cells and macrophages.

**Conclusions:**

Obesity has significant negative effects on lymphatic transport, DC cell migration, and lymph node architecture. Loss of T and B cell inflammatory reactions does not protect from impaired lymphatic fluid transport but preserves DC migration capacity. Future studies are needed to determine how the interplay between diet, obesity, and the lymphatic system modulate systemic complications of obesity.

## Introduction

Obesity results in pathologic changes in virtually every organ system and is a leading cause of morbidity and mortality. It is estimated that nearly 1 in 4 Americans are obese with the incidence rapidly rising in adults and pediatric populations [1]. The cardiovascular system has been a major focus of research on obesity-induced pathology and recent studies have elucidated some of the mechanisms by which obesity promotes vascular injury. However, although the effects of obesity on the cardiovascular system have been intensely studied, there is comparably little known about how dietary changes or adipose accumulation impact the lymphatic system. This is important since the lymphatic system plays a critical role in returning interstitial fluid and lipids to the cardiovascular system. Since the lymphatics are key regulators of inflammation, they may exhibit a primary role in regulating cardiovascular injury in obesity. Therefore, understanding how obesity and dietary changes regulate lymphatic function and immune responses is important and may provide important insights into the pathology of obesity.

The hypothesis that obesity or dietary changes can cause pathological changes in the lymphatic system is supported by several studies. For example, recent reports have shown that obese patients have significantly impaired tissue clearance of macromolecules [2] [3]. In addition, clinical reports have documented spontaneous development of lymphedema, a morbid clinical condition characterized by accumulation of interstitial fluid and localized adipose deposition, in obese patients. Similarly, postoperative weight gain has been shown to increase the risk of lymphedema in patients who undergo lymph node dissection suggesting that the detrimental effects of obesity and surgery are additive [4]. Finally, experimental studies in hypercholesterolemic mice have shown that these animals develop progressive lymphatic dysfunction resulting in tissue swelling, lymphatic vessel leakage, and impaired immune cell trafficking [5]. While all of these studies strongly suggest that obesity impairs lymphatic function, the mechanisms that regulate these effects remain unknown.

The purpose of this study was to identify the pathologic consequences of diet induced obesity (DIO) on the lymphatic system. We show that C57B6-6J mice fed a high fat diet not only become obese over a relatively short period of time, but that these mice also have markedly decreased lymphatic fluid flow and impaired migration of dendritic cells (DCs) to draining lymph nodes. In addition, obese mice have significantly smaller peripheral lymph nodes with loss of normal T and B cell architecture, and loss of gradients of lymph node CCL21 expression. Because previous studies have shown that metabolic syndrome secondary to obesity is dependent on T and B cell inflammation [6], we analyzed the effects of obesity in RAG mice, which are deficient in these cell populations. RAG mice also had significantly decreased lymphatic fluid flow; however, in contrast to wild-type mice, high fat diet feeds in RAG mice did not impair DC migration. Taken together, our findings strongly suggest that DIO results in significant pathologic changes in the lymphatic collecting system and lymph nodes, and that at least some of these changes are dependent on T or B cell inflammatory reactions.

## Materials and Methods

### Diet induced obesity

All experimental protocols were reviewed and approved by the IACUC committee at Memorial Sloan-Kettering Cancer Center. Male C57BL/6J and RAG mice based on the same genetic background (B6.129S7-Rag1tm1Mom/J) were purchased from Jackson Laboratories (Bar Harbor, Maine) and maintained in a temperature and light controlled environment. Previous studies have shown that a diet high in fat (60% Kcal from fat) in these animals reliably results in moderate obesity as compared to animals fed a normal chow or low fat diet and that this model is a useful analog to diet induced (rather than genetic) obesity in humans [7]. Therefore, in order to induce diet-induced obesity (DIO), wild-type and RAG mice were maintained on a high fat diet (60% kcal from fat; W.F Fisher & Son, Inc., NJ) beginning at 6 weeks of age *ad libitum* for 8–10 weeks. Age-matched control male wild-type or RAG mice were maintained on a normal chow diet (13% kcal from fat; Purina PicoLab Rodent Diet 20, W.F Fisher & Son) for the same period of time. At the conclusion of the experiment, animals were weighed using a digital scale (Sartorius, Bradford, MA), and a 12-hour fasting blood draw was performed from the retro-orbital sinus. Serum glucose, cholesterol, and triglyceride levels were analyzed using standard assays (ALX laboratories (New York, NY).

### Analysis of lymphatic function

We used multiple methods to analyze lymphatic transport in order to enable us to visualize dermal lymphatic vessels and to analyze lymph node transport. Microlymphangiography was performed to visualize lymphatic vessels and to analyze the rate of lymphatic fluid transport using our previously published methods [8]. Briefly, fluorescein isothiocyanate (FITC)-conjugated dextran (2,000 kDa, 10 mg/ml, Invitrogen) was injected into the distal tail of mice using a 30 gauge needle under controlled pressure and then visualized at various time points using the Lumar Stereoscope (Carl Zeiss Inc, Peabody, MA). Captured images were analyzed using the Metamorph imaging software (Molecular Devices, Sunnyvale CA) while keeping exposure, gain, and magnification constant. Uptake of FITC-Dextran in the proximal region of the tail was calculated and expressed as the ratio of average pixel intensity of regions distal to the injection site in 5 animals per group.

To confirm our analysis with microlymphangiography and to better analyze three-dimensional structural changes in the lymphatic system, we used a technique recently described by our group using Positron Emission Tomography (PET) and Computed Tomography (CT) imaging [9]. This analysis was performed after intradermal tail injection of fluorodeoxyglucose (18F-FDG) and enables us to trace lymphatic flow along lymph nodes basins. Briefly, mice were imaged prone using the Focus 120 microPET small-animal scanner (Concorde Microsystems Inc.) with an energy window of 350–700 keV and coincidence-timing window of 6 ns. Acquisition began immediately after intradermal injection for as long as 20 minutes. A custom-built restraint device registered PET and CT image data [10]. CT was performed using a microCAT II (ImTek Inc.) scanner operating at 60 kVp and 0.8 mA, with 2-mm aluminum filtration. The fusion montage was arranged in ImageJ (NIH). Three-dimensional rendering was performed using Amira (version 5.0; Visage Imaging GmbH). PET data were corrected for detector non-uniformity, dead time, random coincidences, and physical decay. List-mode data were binned into histograms at durations of 0.5, 1, and 5 min to study different features of 18F-FDG transport and uptake. Images were reconstructed by both maximum a priori and three-dimensional filtered back-projection using a ramp filter with a cutoff frequency equal to the Nyquist frequency into a 128 • 128 • 95 matrix [11]. Images were analyzed using ASIPro (Concorde Microsystems Inc.) with window and level settings adjusted for optimal visualization of lymph nodes in 3–4 animals per group.

We have previously shown that lymphoscintigraphy can be used to accurately analyze lymph node uptake after peripheral injection and is therefore a means of quantifying lymphatic transport to the local lymph node drainage basin. We therefore used a modification of this technique to analyze popliteal lymph node uptake in DIO or control mice [8]. Briefly, 50 μl of technetium Tc^99^m (Tc^99^) labeled sulfur colloid was injected in the distal hind limb (volar palm) and subsequent popliteal lymph node uptake was assessed using an X-SPECT camera (Gamma Medica, Northridge, CA) for 90 minutes. Region-of-interest analysis was performed to derive decay-adjusted activity using ASIPro software (CTI Molecular Imaging, Knoxville, TN). All experiments were performed in a minimum of 5 animals per group.

### Histology and flow cytometry

Back sections were harvested from obese mice and control mice utilizing 5 mm punch biopsy kits (Fray Products Corp, Buffalo, NY). Specimens were briefly fixed in 4% paraformaldehyde (PFA, Affymetrix, Cleveland, OH), placed in 70% alcohol and subsequently paraffin-embedded. Tail sections were harvested for cross-sectional cuts and longitudinal cuts, briefly fixed in PFA and subjected to EDTA (Sigma-Aldrich) for 72 hours for decalcification, after which point sections were placed in 70% alcohol and subsequently paraffin-embedded. We performed immunohistochemistry on tissue sections to localize the expression of CD45 (Rat anti-mouse CD45 mAb (Clone 30-F11), R&D Systems, Minneapolis, MN), LYVE-1 (Goat anti-mouse polyclonal LYVE-1, R&D Systems), and Podoplanin (Syrian hamster anti-mouse monoclonal antibody; Abcam, Cambridge, MA) utilizing a DAB-based development system (DAB, Dako, Carpinteria, CA). Additionally, to co-localize CD45 and LYVE-1 expression, tissue sections were stained for CD45 utilizing a DAB-based development followed by florescent LYVE-1 staining. Images were then overlaid to localize inflammatory cell populations in relation to lymphatic vessels.

Inguinal lymph nodes were harvested and briefly fixed in 4% PFA and embedded in Optimal Cutting Temperature compound (O.C.T. Compound, Tissue Tek, Sakura, Torrance, CA). Tissue sections were then prepared at a thickness of 10 microns using a cryostat (Leica, Buffalo Grove, IL) and mounted on Fisher Plus Slides (Fisher, Pittsburgh, PA). Immunofluorescent staining of lymph nodes was performed using our previous methods [8]. Briefly, lymphatic vessels were identified using LYVE-1 antibodies (R&D Systems, Minneapolis, MN). T cells and B cells were identified using antibodies against CD3 (Rabbit anti-mouse CD3 mAb [ab16669], Abcam) and B220 (Rat anti-mouse B220/CD45R mAb [MAB1217] R&D Systems) respectively. Additionally, we analyzed the expression patterns of CCL21, a critical regulator of cell migration to the lymph node and architectural organization, [12], [13] using a Rat anti-mouse mAb against CCL21 (MAB457, R&D Systems). Negative control sections were incubated with isotype control antibody or secondary antibody alone. Specificity was confirmed using single-stained sections and negative controls. Images were captured using an Axioscope (Carl Zeiss) as previously described [8]). Lymph node lymphatic vessel density was determined using Metamorph™ Offline software and expressed relative to node area (vessels/mm^2^) as previously described [14]. We used a minimum of 5–8 animals per group, per experiment.

In order to determine how DIO regulates the populations of cells in lymph nodes, we performed flow cytometric analysis on inguinal lymph nodes. Single cell suspensions were prepared from inguinal lymph nodes and analyzed using fluorophore-conjugated flow cytometry optimized mouse monoclonal antibodies (EBioscience, San Diego, CA) to identify T-helper cells (CD45^+^, CD3^+^, CD4^+^), cytotoxic T cells (CD45^+^, CD3^+^, CD8), B cells (CD45^+^, B220^+^, CD19^+^), and macrophages (CD45^+^, B220^+^, CD11b^+^). We blocked endogenous Fc receptor binding using Fc block (CD16/CD32) and optimized cytometer settings using splenocytes. Flow cytometry was performed using an LSR II flow cytometer with BD FACSDiva software and data was analyzed using FlowJo Software (Tree Star, Ashland OR). We used 5–7 animals per group for this analysis.

### Whole mount staining

Ear whole mounts were performed as described by Nitschke *et al* [15]. Briefly, mice were sacrificed and ears were harvested after hair removal with Nair (Church and Dwight Co, Princeton, NJ). Ears were subsequently split into 2 halves, followed by blocking in 12% bovine serum albumin (BSA)/phosphate-buffered saline (PBS). Ears were incubated in Lyve-1 (Goat anti-mouse LYVE-1 pAb [AF2125], R&D Systems) for two hours at room temperature followed by secondary antibody incubation (donkey anti-goat Alexa Fluor 594, Invitrogen) at room temperature for 30 minutes. Tissues were then washed in PBS/0.1% Tween-20 and incubated in Rat anti-mouse CCL21 mAb for two hours at room temperature followed by secondary antibody incubation at room temperature for 30 minutes. Tissues were then incubated in DAPI (Sigma-Aldrich) for 30 minutes and mounted with VectaMount AQ (Vector Laboratories, Burlingame, CA) and then imaged using a Zeiss SP5-U confocal microscope (Carl Zeiss). Images were acquired using Zeiss Zen 2010 software (Carl Zeiss) and Imaris version 7.2.3 software (Bitplane) was used for offline image processing. In order to determine colocalization of LYVE-1^+^ cells with CCL21 expression, the fluorescent images were subjected to red, green, blue color separation and the percent overlap of red pixels (CCL21) with green pixels (LYVE-1) was calculated using Metamorph™.

### Dendritic cell migration assay and FITC Painting

We used a modification of the methods outlined by Lim *et al* in order to determine how DIO regulates migration of dendritic cells (DCs) from the periphery to draining lymph node basins [5]. Briefly, the spleens of green florescent protein (GFP) expressing C57B6/6J mice (Jackson Laboratories) were harvested and digested with collagenase D (Sigma-Aldrich, St Louis, MO) at 37°C for 15 minutes with gentle agitation. We then enriched the population of cells for DCs using a magnetic microbead-based positive selection kit for CD11c^+^ cells (Miltenyi Biotech, Gladbach, Germany) according to the manufacturer's recommendations. Isolated cells were resuspended in PBS and injected into the right hindpaw (10^6^ cells per injection) of experimental or control animals, and 36 hours later the right popliteal lymph nodes were harvested and analyzed to determine the number of migrating DCs (GFP^+^/CD11c^+^/MHC-II^high^) using flow cytometry (LSRII; BD Biosciences, San Jose, CA) and Flowjo software (Tree Star, Ashland, OR). Each experiment was repeated in 4–5 animals per group.

We used a well-described FITC painting assay in order to analyze baseline rates of DC migration in wild-type and RAG mice [13]. The backs of wild type and RAG mice were shaved and painted with 8% FITC (type I isomers; Sigma-Aldrich, St. Louis, MO) diluted in a 1∶1 mixture of acetone and dibutylphthalate (Sigma-Aldrich). After 72 hours, mice were sacrificed, and draining LNs were analyzed for migrated DCs (FITC^+^/CD11c^+^/MHC-II^high^) by flow cytometry (LSR II flow cytometer; BD Biosciences, San Diego, CA). The experiment was repeated in 4 animals per group.

### Statistical analysis

The Student’s T-test was used to compare differences between 2 groups while comparison of multiple groups was performed using analysis of variance with post hoc tests (Tukey Kramer) to compare differences between individual groups. Pearson’s coefficient was used to determine the correlation between groups. Data are presented as mean ± standard deviation unless otherwise noted with p<0.05 considered significant.

## Results

### High fat diet results in obesity and peripheral inflammation

**Figure 1:**
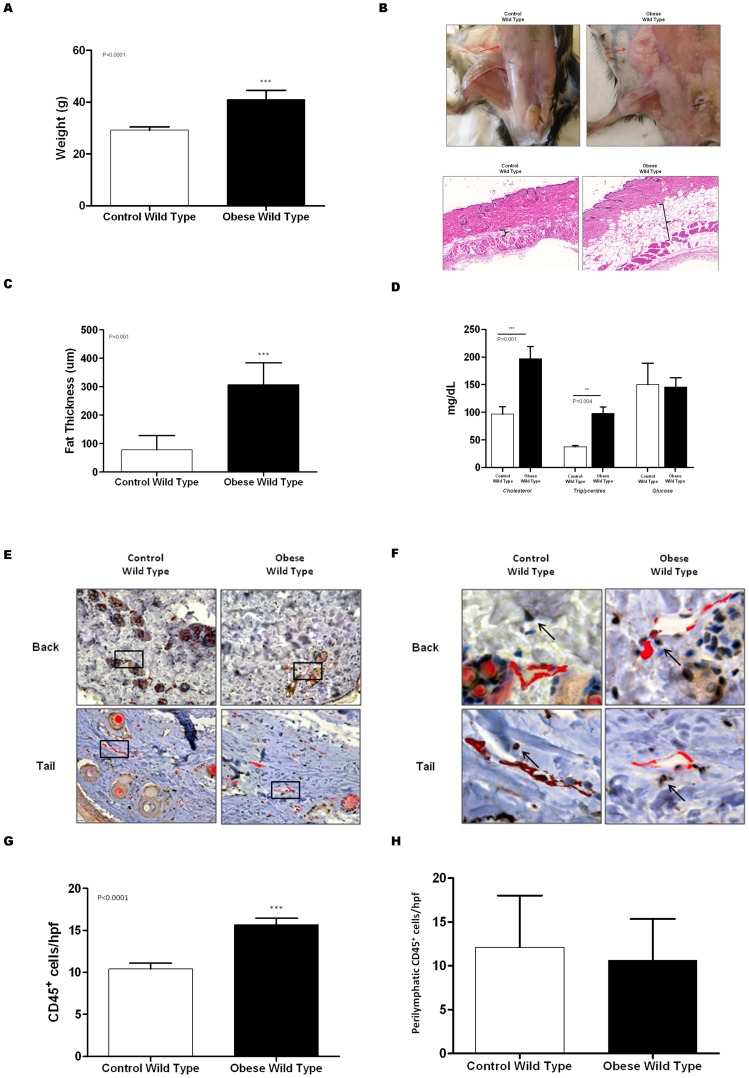
High fat diet results in obesity and subcutaneous tissue inflammation. **A.** Body weight of obese and control mice. **B.** Gross photograph of subcutaneous tissue adipose deposition in control and obese mice (top). Representative photomicrograph of full-thickness back punch biopsy specimens in control and obese mice (20× magnification). Brackets indicate subcutaneous fat deposition. **C.** Mean subcutaneous fat thickness in back punch biopsy of control and obese mice. **D.** Serum cholesterol, triglycerides, and glucose levels in 12 hour fasting blood samples from control and obese mice. **E., F.** Representative low (**E;** 10x) and high (**F;** 40x) power photomicrographs of control and obese mice back punch (upper) and tail (lower) histological sections stained for CD45 (brown) and LYVE-1 (red). Black box in figure E is magnified in F. **G.** Mean number of CD45^+^ cells/hpf (40x) in control and obese mice. **H.** Mean number of peri-lymphatic CD45^+^ cells/hpf (40x) in control and obese mice.

As expected, feeding male C57BL/6J mice a high fat diet for 8–10 weeks resulted in a modest (33%), though significant increase in body weight as compared to control mice (**Figure** **1A**; p<0.0001). Obese mice had significant accumulation of adipose tissues subcutaneously (3-fold increase; p<0.001; **Figure** **1B, C**). In addition, in line with previous reports, we found that obese mice had significantly increased (2-fold) serum levels of total cholesterol and triglycerides as compared to controls (p<0.001). However, DIO had little effect on fasting serum glucose levels (**Figure** **1D**).

Obese mice had mild subcutaneous tissue inflammation as reflected by a 1.5 fold increase in the number of CD45^+^ cells in these regions (all p<0001; **Figure** **1E–G**). However, despite the generalized subcutaneous tissue inflammation, we did not note significant differences in the number of peri-lymphatic CD45^+^ cells when we co-localized CD45 and LYVE-1 (**Figure** **1H**). Indeed, we only noted scattered CD45^+^ cells around individual capillary or collecting lymphatics in both groups when we examined back punch and tail cross-sections.

### Obesity impairs lymphatic flow

**Figure 2:**
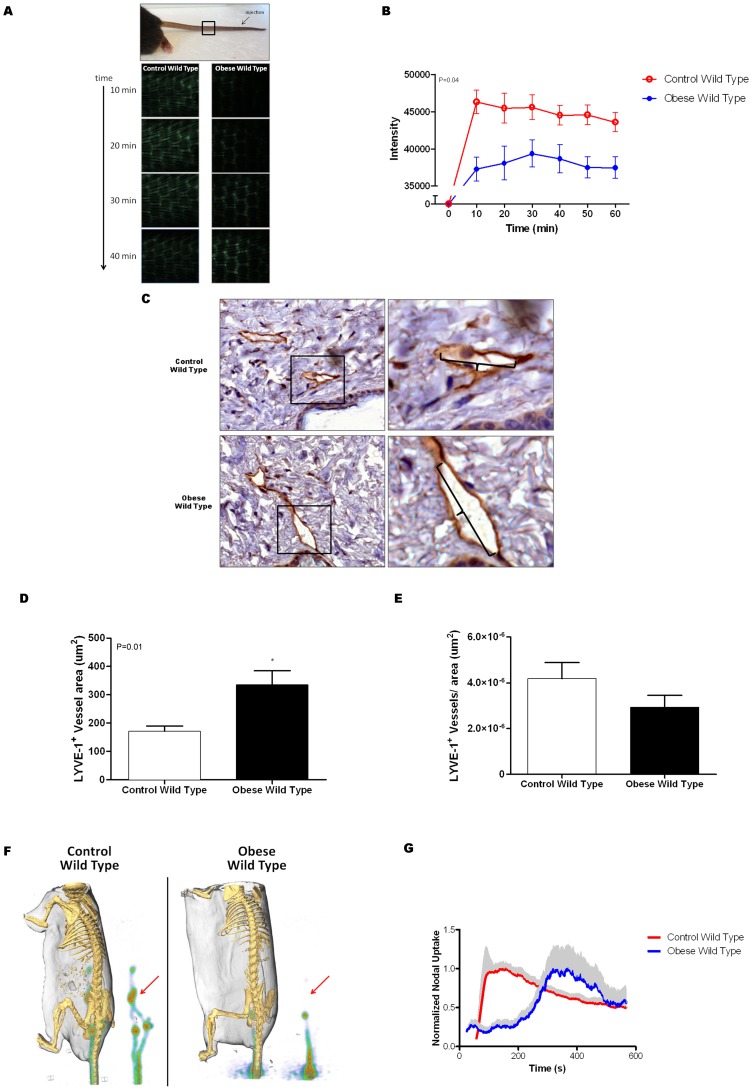
Obesity impairs lymphatic flow. **A., B.** Representative florescent photomicrograph of microlymphangiography (**A**) and quantification of florescence intensity at a fixed distance from the injection site (black box on photograph; **B**) in control and obese mice. The microlymphangiography photographs depict the uptake of florescent labeled macromolecule in the dermal lymphatics of the tail at various time points following injection in the distal tail. Note rapid uptake (10 minutes) and increased florescence intensity in control mice as compared with obese mice. This difference is quantified in figure **B** in 5 animals per group. **C.** Representative low power (20x; upper) and higher power (40x; lower) photomicrographs of back punch tissue sections from control and obese mice stained for LYVE-1 (brown). Boxes in upper panel represent area magnified in high power views. Brackets denote diameter of the lymphatic vessels. **D.** Mean lymphatic vessel area in control and obese mice. **E.** Mean number of LYVE-1^+^ lymphatic vessels/hpf in back punch biopsies of control and obese mice. **F., G.** Representative PET-CT of control and obese mice demonstrating lymphatic chain after distal tail injection of 18F-FDG. The lymphatic chain is also shown separately for greater detail. Arrow denotes location of para-aortic lymph nodes. Mean normalized sacral node uptake ± SEM (gray shading) of 18F-FDG over time in control and obese mice.

We next used microlymphangiography in order to visualize dermal lymphatic vessels and analyze lymphatic fluid flow. Interestingly, we found that lymphatic transport of FITC-dextran labeled colloid to the proximal portion of the tail in obese mice was not only markedly delayed (3-fold; p<0.01), but also of lower intensity as compared to controls suggesting that both the uptake and transport of interstitial fluid in obese mice is decreased relative to controls (**Figure 2A, B**). This finding was supported by the fact that LYVE-positive capillary lymphatic vessels in DIO mice were significantly more dilated as compared with controls; a finding that has been shown to be correlated with lymphatic fluid stasis (**Figure 2C, D**) [16]. However, we did not find differences between groups in the total number of LYVE-positive dermal capillary lymphatics in skin harvested from the back (**Figure 2E**). Additionally, we did not observe differences in the number of Podoplanin-positive lymphatic vessels between control and obese mice (**Figure E, F in File S1**).

We have previously shown that 18F-FDG, a large radio labeled macromolecule, preferentially travels via the lymphatic circulation thus enabling us to analyze lymphatic fluid transport and lymph node uptake using PET-CT imaging [16]. Similar to our findings with microlymphangiography, we found that 18F-FDG uptake in the sacral lymph nodes (the first drainage basin after distal tail injection) was markedly delayed in obese mice as compared to controls (**Figure** **2F, G**). Thus, in contrast to controls in which we rapidly (<100 seconds) noted peak uptake of 18F-FDG in the sacral lymph nodes, uptake of this molecule in DIO mice was markedly delayed (nearly 400 seconds after injection). More importantly, we found that in contrast to control mice, DIO mice demonstrated minimal uptake of 18F-FDG in the para-aortic and portal lymph nodes (the next two draining basins). Taken together, our results suggest that DIO results in decreased interstitial fluid flow, decreased lymphatic drainage to local lymph node basins, and decreased lymphatic transport between adjacent lymph node basins.

### Obesity impairs dendritic cell migration in wild-type but not RAG mice

Previous studies have shown that inflammation is a critical regulator of pathologic responses to obesity including metabolic syndrome, atherosclerosis, and cancer [17]. More recent studies have shown that T cell and B cell inflammation are key regulators of this process [6], [7], [18], [19]. In addition, our lab and others have shown that T cells negatively regulate lymphangiogenesis and lymphatic function [20], [21]. Therefore, in order to determine how T/B cell inflammatory reactions regulate the pathologic consequences of obesity on the lymphatic system, we fed RAG mice (lack mature T and B cells) either a high fat (HFD) or normal chow diet and analyzed interstitial fluid flow and dendritic cell migration.

In line with previous reports, RAG mice fed a HFD (obese RAG) weighed on average 15 g more than control mice fed a normal chow diet (**Figure** **A in File** **S1**; p = 0.0006). In addition, similar to wild-type mice, obese RAG mice had modest, though significant increases in total serum cholesterol as compared to controls (p = 0.02); however, triglycerides and glucose were relatively unchanged (**Figure** **B in File** **S1**).

**Figure 3:**
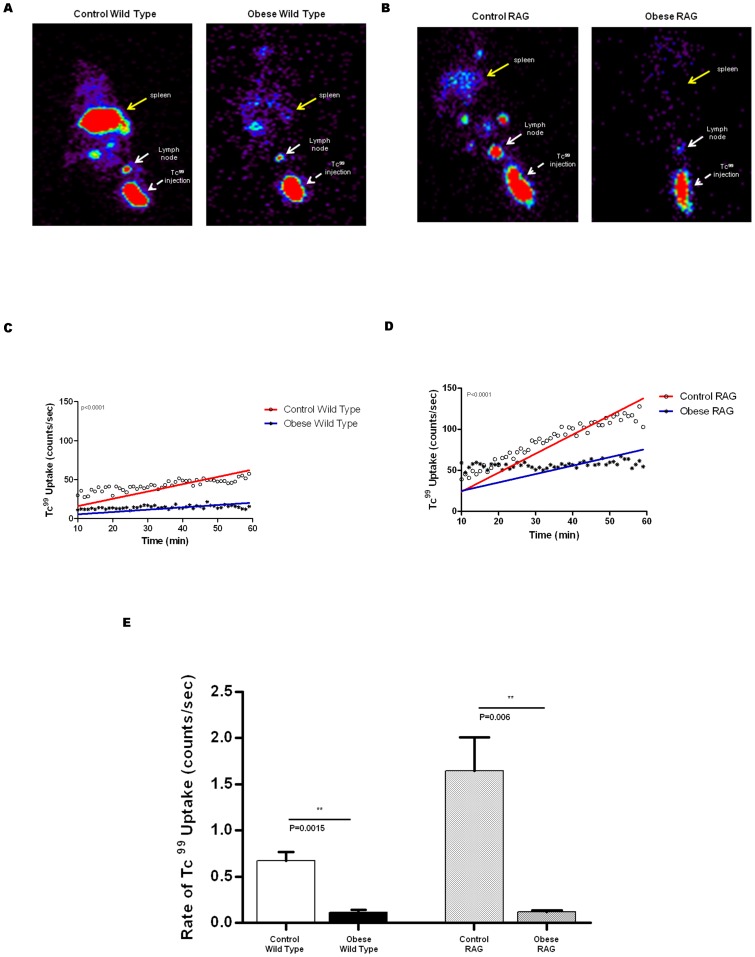
Obesity decreases lymph node uptake. **A., B.** Tc^99^ lymphoscintigraphy in wild-type (**A**) and RAG (**B**) mice. Yellow arrow denotes spleen. White arrow is the lymph node; arrow head denotes Tc^99^ injection site. **C., D.** Mean Tc^99^ uptake in wild-type (C) and RAG (B) control and obese mice. **E.** Mean rate of Tc^99^ uptake in control and obese wild-type and RAG mice.

We next used lymphoscintigraphy with Tc^99^ labeled sulfur-colloid to analyze lymphatic function in obese and lean wild-type/RAG mice since we have previously shown that this technique is highly sensitive and capable of demonstrating even minor differences in lymphatic function [8], [22], [23]. This increased sensitivity is due in part to the fact that Tc^99^ uptake in the primary lymph node basin, in contrast to 18F-FDG, is cumulative and decay adjusted, enabling us to control for minor differences in injection techniques. Heat map analysis of popliteal lymph nodes after hind limb injection demonstrated markedly decreased lymph node TC^99^ uptake in obese wild-type and RAG mice as compared to their respective lean controls (**Figure** **3A, B**). This finding was supported by decreased Tc^99^ uptake in the spleen of obese wild-type and obese RAG mice as compared with their controls suggesting that return of interstitial fluid to the lymphatic circulation is delayed in obese animals. Quantification of popliteal Tc^99^ uptake, similar to our findings with microlymphangiography and 18F-FDG PET-CT scanning, demonstrated that *both* the rate and cumulative uptake of Tc^99^ in popliteal lymph nodes of obese wild-type and RAG mice is significantly decreased as compared to controls (**Figure** **3C–E**). These experiments showed that control wild-type and RAG mice had a steady increase in Tc^99^ uptake in their popliteal lymph node during the course of the experiment as evidenced by the increasing slope of the best-fit line. In contrast, obese wild-type, and to a lesser extent RAG, mice demonstrated an initial low uptake early in the course of the experiment but total uptake did not substantially increase over time.

Interestingly, these studies demonstrated baseline differences between wild-type and RAG mice in Tc^99^lymph node uptake. For example, as shown in **Figures 3C and 3D**, it was evident that RAG mice (both obese and lean) had a higher (nearly 2-fold increased) baseline popliteal lymph node uptake of TC^99^ as compared with wild-type mice. In addition, while the total uptake of Tc^99^ in obese RAG mice popliteal lymph nodes at 90 minutes was decreased approximately 2.4 fold when compared with lean RAG controls, this difference was less pronounced than the decreased total uptake we noted in wild-type DIO mice compared with their lean controls (3.3 fold decrease).

**Figure 4:**
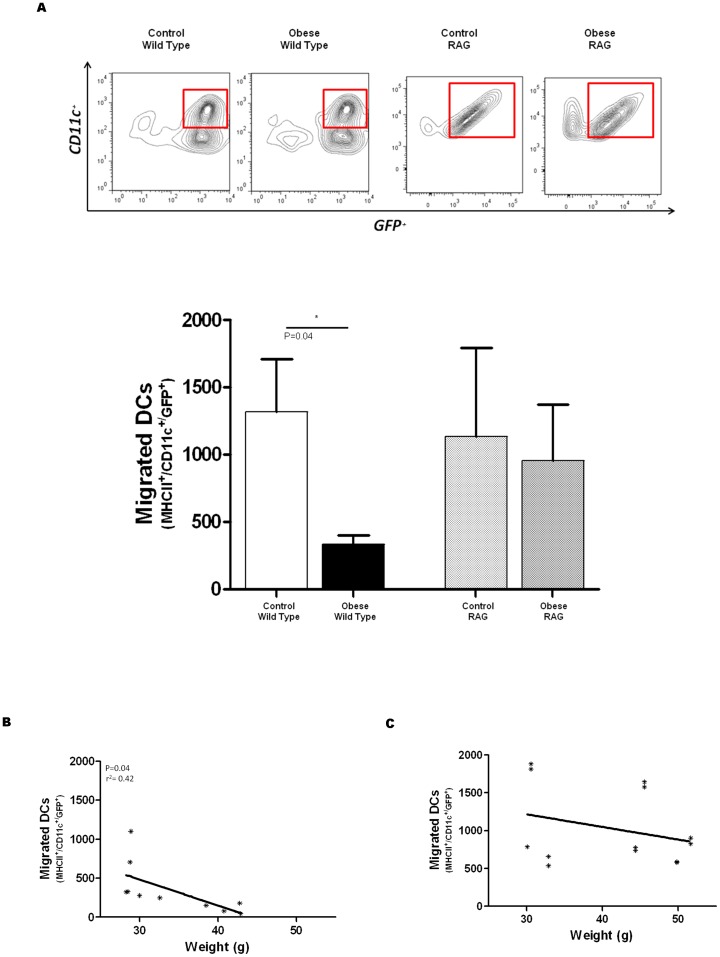
Obesity impairs dendritic cell migration in wild-type but not RAG mice. **A.** Representative flow diagram (top) and quantification (bottom) of migrated DCs (MHCII^high^/CD11c^+^/GFP^+^) in control and obese wild-type and RAG mice. **B., C.** Correlation between migrated DCs and weight in wild-type (**B**) and RAG (**C**) mice.

After finding that lymphatic flow was diminished in obese mice, we next sought to determine if dendritic cell migration is also impaired in the setting of obesity. In order to test this hypothesis, we isolated leukocytes from splenic homogenates of syngeneic GFP^+^ mice, enriched this population for dendritic cells (DC) using magnetic selection (50-fold enrichment; **Figure C in File S1**), and injected them into the hind paw of obese or lean wild-type and RAG mice. Analysis of popliteal lymph nodes for GFP^+^ DCs (i.e. MCHII^high^/CD11c^high^/GFP^+^) 36 hours after injection demonstrated that obese wild-type mice had markedly decreased DC migration to the lymph node from the injection site as compared with their lean controls (5 fold decrease; p<0.035; **Figure 4A**). In addition, we found a statistically significant negative correlation between weight and the number of migrated GFP^+^ DCs in wild-type mice such that the heaviest animals tended to have the fewest number of GFP^+^ DCs in their lymph nodes (**Figure 4B**; p<0.05; r = 0.42). In contrast, although the number of migrated GFP^+^ DCs was slightly decreased in obese RAG mice as compared to lean RAG controls, this difference did not reach statistical significance (**Figure 4A**). In addition, in contrast to wild-type mice, we did not find a correlation between DC migration and weight in RAG mice (**Figure 4C**).

We next used FITC skin painting to determine if the higher baseline rates of lymph node TC^99^uptake we observed in RAG mice relative to wild-type mice translate to intrinsic differences in the rates of DC migration in these animals. However, comparison of lean wild-type with lean RAG mice did not demonstrate significant differences in the rates of DC transport to the draining lymph nodes (**Figure D in File S1**) suggesting that higher baseline lymphatic transport rates of RAG mice is likely not the mechanism by which DC migration potential is preserved in obese RAG mice.

Taken together, the findings in this portion of our study suggest that T and B cell inflammatory reactions in obese animals are not necessary for impaired lymph node interstitial fluid uptake but that these immune responses do have a negative effect on migration of DCs to the lymph node.

### Obese mice have smaller lymph nodes with fewer lymphatic vessels

**Figure 5:**
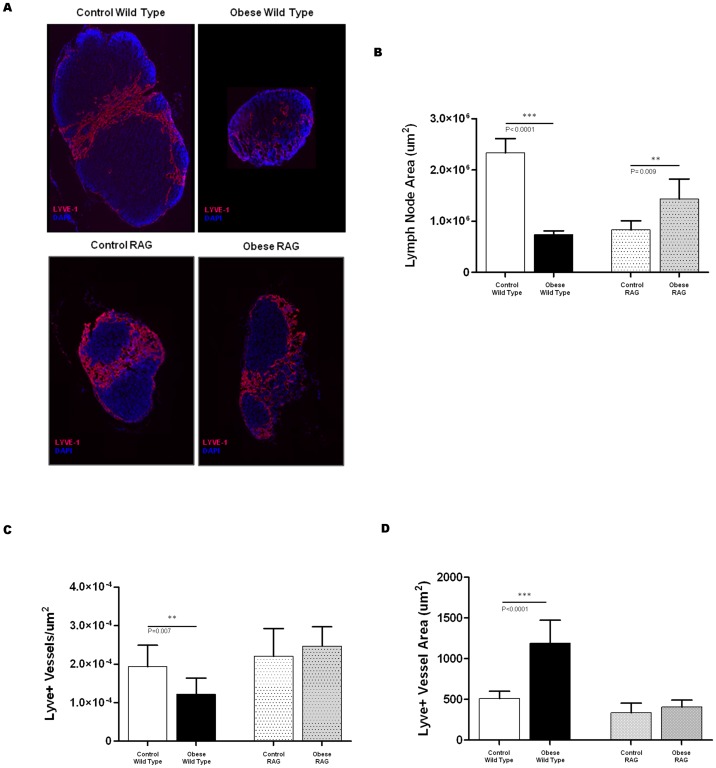
Obese mice have smaller lymph nodes with fewer lymphatic vessels. **A.** Representative immunofluorescent staining (LYVE-1 =  red; DAPI  =  blue) of inguinal lymph nodes (2× magnification) harvested from control (left panels) and obese (right panels) wild-type and RAG mice. **B.** Mean lymph node area of inguinal lymph nodes harvested from control/obese wild-type and RAG mice. **C.** Mean number of lymphatic vessels per unit area in inguinal lymph nodes of control/obese wild-type and RAG mice. **D.** Mean lymphatic vessel area in the inguinal lymph nodes of control/obese wild-type and RAG mice.

Given that we found that obese mice had diminished lymphatic flow and DC migration to regional lymph nodes, we next sought to determine if these effects translate to structural changes in lymph nodes. Consistent with previous reports on mesenteric lymph nodes in obese mice, [24] we found that inguinal lymph nodes of DIO wild-type mice were significantly smaller in size (3.4-fold) as compared to lean control mice (**Figure 5A, B** **;** p<0.0001). In contrast, inguinal lymph nodes harvested from obese RAG mice were nearly 1.7 fold (p = 0.009) larger than their respective controls although the baseline size of the lymph nodes in the lean control RAG mice was significantly smaller than lean control wild-type mice.

Analysis of lymphatic vessel density and size further demonstrated differences in response to obesity in wild-type and RAG mice. Consistent with our observation of decreased interstitial fluid flow to the regional lymph nodes in obese wild-type mice, we found that these mice also had significantly decreased number of LYVE-1^+^ lymphatic vessels (1.6 fold) in their lymph nodes (**Figure 5C** **;** p = 0.007). In contrast, obese RAG mice had similar numbers of lymph node lymphatic vessels as compared to RAG mice fed a NCD. Also consistent with lymphatic dysfunction, we found that obese wild-type mice had mildly dilated (2.3 fold) lymph node lymphatics as compared to controls (**Figure 5D**; p<0.0001). However, similar to our observation with the total number of lymph node lymphatic vessels, we found that obesity did not result in dilatation of these vessels in RAG mice.

### Obesity causes changes in the architecture and cellular composition of lymph nodes

**Figure 6:**
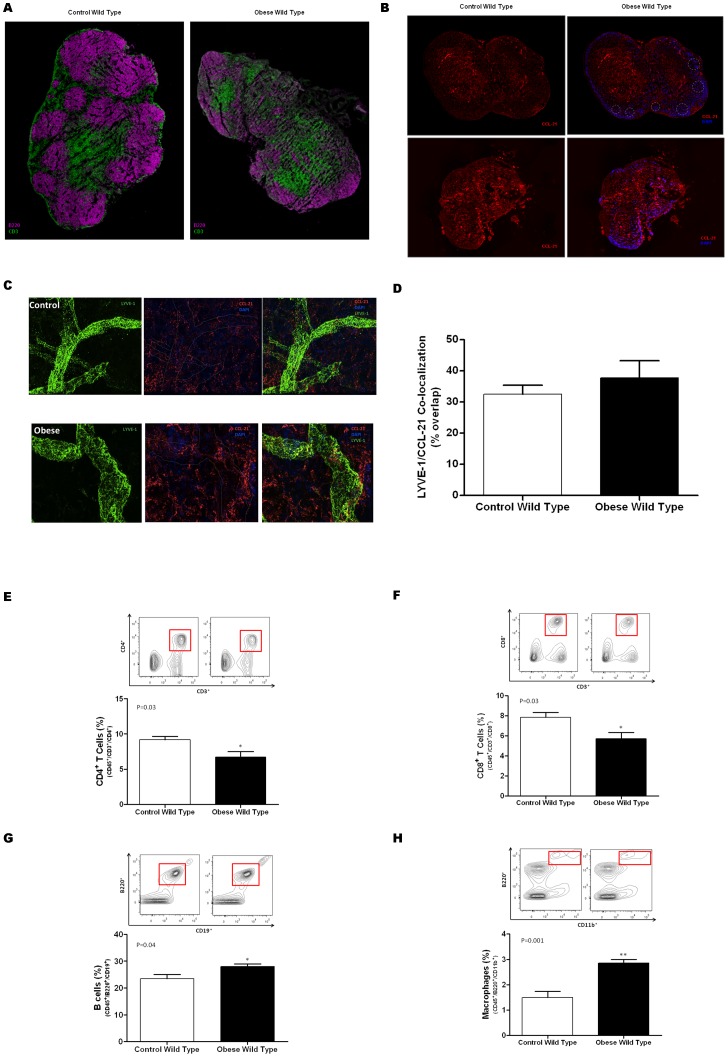
Obesity causes changes in the architecture and cellular composition of lymph nodes. **A.** Representative florescent micrograph (2.5× magnification) of inguinal lymph nodes harvested from control and obese mice and stained for B cells (B220; purple) and T cells (CD3; green). Note loss of follicular pattern and disorganization in obese mice. **B.** Representative florescent micrographs (2.5x) of inguinal lymph nodes harvested from control (left panels) and obese (right panels) wild-type mice stained for CCL21 (red) and DAPI (blue). CCL21 expression is shown on top panels with DAPI overlay on the bottom. Dotted circled represent follicular regions. Note loss of CCL21 expression gradients in obese animals. **C.** Representative whole mount immunofluorescent staining (Green  =  LYVE-1; Red  =  CCL21; Blue  =  DAPI) of ear sections from control (top) and obese (bottom). Sections are shown at 63× magnification. LYVE-1 staining is shown on the left; CCL21 is the middle panel; overlay is shown on the right panels. Dashed lines in the middle panel is the region of LYVE-1 vessel. **D.** Mean area of LYVE-1/CCL21 co-localization (presented as a percentage of LYVE-1 area overall) in control and obese mice. **E., F. G., H.** Flow cytometry analysis of T-helper cells (CD45+/CD3+/CD4+; **E**), cytotoxic T cells (CD45^+^/CD3^+^/CD8^+^; **F**); B cells (CD45^+^/B220^+^/CD19^+^; **G**), and macrophages (CD45^+/^/B220^+^/CD11b^+^; **H**). Mean percentage of cells from 4–5 animals per group is presented.

In order to further assess the structural changes seen in lymph nodes of obese mice, lymph nodes were stained for T and B cell populations. Consistent with previous reports on the effects of lymphatic fluid flow on lymph node architecture, we found that lymph nodes harvested from obese wild-type mice had markedly abnormal T cell and B cell distribution with loss of follicle organization as compared with controls (**Figure 6A**). Because the distribution of T and B cells zones in the lymph node is regulated by gradients of CCL21 expression, we analyzed the expression patterns of CCL21 in the obese and control animals. As expected, we noted that CCL21 was predominantly expressed in T cell zones of lymph nodes harvested from lean control wild-type mice (**Figure 6B**). In contrast, consistent with our observation of abnormal T and B cell distribution in obese mice, we found that the gradients of CCL21 expression were lost resulting in scattered and diffuse expression of CCL21. Interestingly, after analyzing whole mount ear sections that were co-lozalized for CCL-21 and LYVE, no differences were observed in the patterns of peripheral CCL-21 expression between obese and wild type mice (Figure 6 C, D).

To determine if obesity also cause changes in the populations of cells in lymph nodes, we performed flow cytometry on single cell suspensions to analyze the relative percentages of T cells, B cells, and macrophages. This analysis demonstrated that obese mice had modest, though significant decreases in the percentage of CD4^+^ (**Figure 6E**; p = 0.03) and CD8^+^ T cells (**Figure 6F**; p = 0.03). In contrast, the percentage of B cells and macrophages in obese mice were increased as compared to controls (**Figure 6G, H**).

## Discussion

We have shown that diet induced obesity (DIO) in mice results in significant impairment of the lymphatic system as reflected by decreased lymphatic flow, changes in lymph node architecture, and impaired dendritic cell migration. We chose the DIO model rather than genetic models of obesity (e.g. db/db or ob/ob mice) since the cause of obesity in the vast majority of patients is dietary excess rather than genetic abnormalities. Therefore, the DIO model will be useful in future studies since altering dietary fats or pharmacologic interventions will enable us to determine if the negative consequences of obesity on the lymphatic system are reversible. In addition, the DIO model is valuable for our analysis since changes in serum glucose levels in these mice, in contrast to genetic models of obesity, are mild [25]. This is important since significantly increased serum glucose levels in conjunction with obesity could be a potential confounding factor in the analysis of lymphatic function as evidenced by prior studies demonstrating impaired lymphangiogenesis and function in diabetic mice [26]. Finally, the DIO model is more useful for analysis of obesity induced inflammation and lymphatic dysfunction than genetic models since the former (e.g. Ob/Ob mice) are known to have defects in cellular immunity and thymocyte apoptosis [27].

Using a variety of techniques we found that DIO results in impaired lymphatic transport in dermal lymphatics and drainage to the regional lymph nodes. We specifically used multiple imaging modalities (some of which utilize radioactive tracers) to minimize visualization artifacts and confirm that our findings were not secondary to changes in the tissue thickness or conductivity due to obesity. For example, we used not only microlymphangiography, but also technetium 99 lymphoscintigraphy and FDG-PET imaging. Importantly, it is highly unlikely that the depth of penetration would be altered in radioactive tracers. In addition, the fact that all these different modalities had very similar patterns of lymphatic transport and were confirmatory of our findings with microlymphaniography was highly encouraging and suggests strongly that the changes we noted were not merely an artifact but rather a biologic phenomenon.

Our findings are supported by recent clinical studies in obese patients. For example, in a study comparing obese and lean patients, Arngrim *et al* reported decreased lymphatic washout from subcutaneous adipose depot of obese patients [2]. Similarly, Greene *et al* recently reported that massively obese patients have impaired lymphatic drainage by lymphoscintigraphy and spontaneous onset of lymphedema [3]. This finding is supported by previous studies on genetic models of cholesterol metabolism (APO E^−/−^ mice) demonstrating that high fat diets in these animals results in similar acquired defects of the lymphatic system [5]. Interestingly, we also noted that DIO mice had moderate, though significant increases in serum levels of total cholesterol and triglycerides as compared with controls suggesting that this may be a potential mechanism by which lymphatic injury occurs in obesity. However, although we do not have definitive proof, this hypothesis is likely not valid since the levels of cholesterol/triglycerides in our DIO mice were significantly lower than those reported in APO E^−/−^ mice based on the same background (C57B6). Thus, while Lim, *et al* reported total serum cholesterol levels ranging between 1000–2000 mg/dl in APO E^−/−^ mice fed a normal chow or high fat diet, respectively, they only found significant decreases in lymphatic conductance in animals fed a high fat diet (i.e. cholesterol of 2000mg/dl) suggesting that the effect of serum cholesterol on lymphatic function is dose dependent. In our study, mice fed a high fat diet had a total serum cholesterol level of *200* *mg/dl* (10 fold lower than APO E^−/−^ mice) thus making it unlikely that deficits in lymphatic drainage were a consequence of mildly increased serum cholesterol. This concept suggests that pharmacological interventions designed to decrease serum cholesterol may also be minimally helpful in obese patients who do not have genetic abnormalities in cholesterol metabolism.

We used the DIO model in RAG mice to test the hypothesis that T and B cell inflammation resulting from obesity contributes to lymphatic dysfunction. This hypothesis was based on recent literature demonstrating that T cells have important anti-lymphangiogenic roles and can regulate lymphatic function. For example, Kataru et al demonstrated that T cell inflammation in lymph nodes and resultant expression of interferon gamma significantly inhibited inflammatory lymph node lymphangiogenesis [21]. Similarly, our lab has recently shown that T cells regulate the expression of anti-lymphangiogenic cytokines during wound repair and in response to lymphatic fluid stasis [20], [22], [23]. Our finding that RAG mice, similar to wild-type obese mice, had impaired lymphatic uptake of Tc^99^ is therefore important and suggests that lymphatic transport defects that develop in response to HFD induced obesity are independent of T cell or B cell inflammation. However, it is likely that the effects of obesity-induced T/B cell inflammation on the pathological responses of the lymphatic system are complex. This hypothesis is based on previous findings demonstrating that T cell inflammation is necessary for, and precedes macrophage inflammation, of visceral fat [7], [18]. Additionally, T cell differentiation to a Th2 phenotype and T regulatory cell type is protective of obesity-induced inflammation and decreases metabolic syndrome [6], whereas CD8 cells and interferon expressing T cells in contrast, worsen metabolic syndrome and glucose intolerance [7]. Thus, additional work is required to determine how inflammatory responses in obesity regulate lymphatic function.

An interesting finding of our study was that obese mice had significantly smaller lymph nodes, abnormal cellular distribution, decreased relative numbers of T cells, and increased relative numbers of B cells and macrophages as compared with controls. These findings are supported by those of Kim *et al* who found that DIO results in decreased size of mesenteric lymph nodes and reductions in the number of T cells [24]. However, in contrast to our study, Kim *et al* found that DIO also resulted in decreased number of B cells in the mesenteric lymph nodes and hypothesized that T/B cell apoptosis is induced in obesity possibly as a result of exposure to free fatty acids and resultant oxidative stress.

Our findings of decreased lymph node size and architecture are also supported by recent work reported by Thomas *et al* using K14-VEGF-R3-Ig mice [28]. These mice express a soluble VEGF-R3 antibody in the skin and, as a result, have severely hypoplastic dermal lymphatic vessels and decreased lymphatic transport. Similar to our study, Thomas *et al* reported markedly decreased lymph node size and abnormal architecture (B cells scattered among T cells rather than arranged in a follicular pattern) and suggested that lymphatic fluid flow to the lymph node has important regulatory effects on lymph node homeostasis. In addition, similar to our study, the authors reported that the expression patterns of CCL21 was abnormal in K14-VEGF-R3-Ig mice. Taken together, our findings and those of Thomas *et al* support the hypothesis that lymphatic flow to the lymph node is a critical regulator of lymph node architecture and morphology. This hypothesis is further supported by previous studies demonstrating that the expression of CCL21 by lymph node reticular cells is regulated by fluid shear and lymph node interstitial flow [12]. It is likely, however, that multiple mechanisms regulate CCL21 expression in these circumstances since it is known that impaired steady state dendritic cell migration to lymph nodes also results in abnormal T/B cell distribution by regulating T zone fibroblastic reticular cell expression of CCL21 and VEGF [13]. Putative changes in VEGF expression are supported by our findings of decreased lymph node lymphangiogenesis and dilated lymphatics. Thus, it is likely that the architectural disturbances we noted in obese mice is multifactorial.

We found that obesity in wild-type animals markedly decreased dendritic cell migration to regional lymph nodes. Interestingly, using APO E^−/−^ mice fed a HFD, Lim *et al* also found similar impairments in DC trafficking [5]. However, in contrast to our study these authors did not find significant disturbances in wild-type mice fed a HFD. This disparity may be due to differences in diets in our respective studies. Importantly, similar to most reports on DIO in C57B6/J mice, we utilized a diet very high in fat (60%) as compared to a normal chow diet of 13% fat [6]. In contrast, Lim *et al* defined HFD as chow containing 21% milk fat and NCD as 5% milk fat. This diet seemingly did not appear to induce significant subcutaneous fat deposition or obesity, although differences in weight were not noted. In addition, in our study we utilized a strain of C57B6 mice (6/J) that are more prone to diet induced obesity where as the subtype used by Lim *et al* was not reported (other than C57B6). Thus, some of the reported differences likely reflect strain differences as have been previously reported [29].

Although obesity caused impairments in dendritic cell migration in obese wild-type mice, DC migration was relatively preserved in obese RAG mice. This preservation of immune function is likely not due to baseline differences in the rates of DC migration in wild-type or RAG mice since we found no differences using an FITC painting assay. However, analysis of lymphatic transport using Tc^99^ lymphoscintigraphy demonstrated that the total lymph node uptake was markedly higher in RAG mice (both lean and obese) as compared to wild-type animals. This finding suggests that T and B cell function, both in normal and obese animals, may regulate lymphatic fluid transport and lymph node uptake. This hypothesis is supported by our finding that, in contrast to obese wild-type animals, lymph node lymphangiogenesis was not decreased in obese RAG mice. This hypothesis is also supported by recent mice studies demonstrating that T cells negatively regulate lymphangiogenesis during inflammation and wound repair [21].

The finding that obese RAG mice have preserved DC migration capacity suggests that T and B cell inflammatory reactions secondary to obesity may play a role in this response, however, future experiments are needed to further dissect this observation.

In conclusion, we have shown that diet induced obesity has significant negative effects on the lymphatic system regulating lymphatic transport, lymph node architecture, and dendritic cell migration. These changes may be in part due to T or B cell inflammation induced by obesity. These findings are important since the lymphatic system is an important regulator of both cardiovascular fluid transport and inflammatory responses suggesting that diet induced changes in the lymphatic system may modulate the pathology of obesity.

## Supporting Information

File S1 **Contains: Figure A:** Mean weights of control and obese RAG mice. **Figure B:** Mean 12 hour fasting serum cholesterol, triglyceride, and glucose concentration in control and obese RAG mice. **Figure C:** Representative flow cytometry demonstrating enrichment of DCs after magnetic bead selection. **Figure D:** Mean number of migrating DCs in lean wild-type and RAG mice 36 hours after FITC skin painting. **Figure E:** Representative low power (20x; left) and higher power (40x; right) photomicrographs of back punch tissue sections from control and obese mice stained for Podoplanin (brown). Boxes in right panel represent area magnified in high power views. **Figure F:** Mean number of Podoplanin^+^ lymphatic vessels/area in back punch biopsies of control and obese mice.
(ZIP)
